# Ingestion of *Artemisia argyit* essential oil combats *Salmonella pullorum* infections by altering gut microbiota composition in chicks

**DOI:** 10.1186/s13567-025-01527-7

**Published:** 2025-05-06

**Authors:** Linlin Ding, Kaige Qi, Yutong Zhou, Qingjie Li, Minda Liu, Na Hu, Jianfeng Wang, Jiazhang Qiu, Xuming Deng, Lei Xu

**Affiliations:** 1 State Key Laboratory for Diagnosis and Treatment of Severe Zoonotic Infectious Diseases, Key Laboratory for Zoonosis Research of the Ministry of Education, Institute of Zoonosis, and College of Veterinary Medicine, Jilin University, Changchun, 130062 China; 2 Research Center of Traditional Chinese Medicine, The Affiliated Hospital to Changchun University of Chinese Medicine, Changchun, 130021 China

**Keywords:** *Artemisia argyit* essential oil, *Salmonella pullorum*, chick, *Lactobacillus reuteri*, gut microbiota

## Abstract

Pullorum disease, caused by *Salmonella pullorum* (*S. pullorum*), is a highly contagious illness affecting the poultry industry*.* Emerging evidence suggests that *Artemisia argyit* essential oil can influence the composition of gut microbes in the host, thereby promoting overall health. However, the specific mechanisms by which *Artemisia argyit* essential oil modulates gut microbiota to combat *S. pullorum* infection remains unclear. This study explored the effectiveness of various doses of *Artemisia argyit* essential oil in preventing *S. pullorum* infection in chicks. Our findings indicate that consuming this essential oil can mitigate the intestinal mucosal barrier damage and excessive inflammatory response caused by *S. pullorum,* as well as reverse the weight loss seen in infected chicks. Additionally, chicks that received faecal microbiota transplantation (FMT) from the gut microbiota of *Artemisia argyit* essential oil donors exhibited notable recovery from *S. pullorum* infections. This suggests that the observed protection may be linked to the modulation of gut microbiota. Furthermore, 16S rRNA sequencing revealed an increased abundance of *Lactobacillus reuteri* (*L. reuteri*), which along with the activation of Wnt/β-catenin pathways, played critical roles in the enhanced health of *S. pullorum*-infected chicks treated with *Artemisia argyit* essential oil. In summary, these findings highlight that the dietary inclusion of *Artemisia argyit* essential oil promotes the intestinal enrichment of *L. reuteri*, offering a promising strategy for the treatment and prevention of pullorum disease in chicks.

**Supplementary Information:**

The online version contains supplementary material available at 10.1186/s13567-025-01527-7.

## Introduction

The chick industry has experienced rapid growth over the past half-century, becoming the second-largest breeding industry in China [1]. This growth is largely due to the industry’s short feeding cycles, and its ability to provide energy, protein, and essential micronutrients to humans. However, high-density feeding may lead to immune stress or disease in chicks, which negatively impacts their growth performance and the quality of their meat [2].

Newly hatched chicks are particularly susceptible to *Salmonella pullorum* (*S. pullorum*). Infections can result in symptoms such as lethargy, loss of appetite, intestinal damage, and high mortality rates [3, 4]. Furthermore, *S. pullorum* can persist in the macrophages of the infected chicks throughout their lives, posing a risk of transmission to offspring and causing various health issues [5].

For decades, antibiotics have been used effectively to prevent and treat pathogenic infections in chicks. However, the long-term use of antibiotic growth promoters (AGP) disrupts the balance of gut microbiota, leads to antibiotic drug residues, and fosters the development of drug-resistant bacteria, which poses serious risks to public health and contributes to environmental pollution [6, 7].

In contrast, Traditional Chinese Medicine (TCM) offers promising alternatives with several advantages, including ready availability, low toxicity, minimal clinical side-effects, significant structural diversity, and biocompatibility, which solidifies its efficacious anti-infection effects [8, 9]. Growing evidence suggests that gut microbiota can serve as a reference for guiding the clinical application of TCM [10]. TCM helps establish microbiome homeostasis, control pathogen proliferation [11], and improves meat quality and immunity in poultry production [12].

Among the various natural products derived from TCM, essential oils have gained significant attention due to their effective properties. Studies have shown essential oils can significantly reduce colonisation of *Clostridium perfringens* and *Campylobacter jejuni* in chicks [13–15]. One notable example is *Artemisia argyit,* a traditional herbal medicine in China for over one thousand years. The essential oil extracted from *Artemisia argyit* (AAEO), commonly known as mugwort (Chinese name: aicao), contains a variety of aromatic compounds that offer pharmacological properties, including anti-inflammatory and antioxidant effects [16].

Collectively, essential oils present great potential in combating bacterial infections in chickens. However, there is still limited knowledge regarding the effectiveness of *Artemisia argyit* essential oil in modulating gut microbiota homeostasis to alleviate bacterial infections in chicks.

The intestinal microbiota, often referred to as the “invisible organ”, plays a crucial role in improving the digestive system and maintaining gastrointestinal homeostasis [17]. This population of microorganisms residing in the intestine significantly impacts both growth performance and host health [18]. It is well established that intestinal microorganisms influence host physiology in various ways. In cases of pathogen infection, an imbalance in gut microbiota can lead to decreased immune function, inflammatory diseases, and impaired intestinal function [19]. Therefore, establishing a stable gut microbiome in chicks is essential for combating bacterial infections [20].

Recent studies have focused on the effects of probiotics on chick intestinal development, microstructure and microbiological characteristics [21, 22]. Among the major probiotics found in the intestinal tracts of chickens, *Lactobacillus reuteri* (*L. reuteri*) has garnered much attention for its role in restoring growth performance and preventing intestinal damage in chicks [23–25]. Additionally, *L. reuteri* supplementation can help mitigate the adverse effects of *Staphylococcus aureus*-induced mastitis in mice by enhancing intestinal barrier function [26].

Encouragingly, previous research has shown that *Artemisia argyi* polyphenols can alleviate dextran sulfate sodium (DSS)-induced ulcerative colitis by altering gut microbiota [27]. In particular, *Artemisia argyi* polysaccharide treatment enhances the abundance of *Lactobacillus* in the gut microbiota of mice [28, 29]. These findings suggest that extracts from *Artemisia argyi* possess probiotic properties that influence the composition and metabolism of the microbiota, thereby improving intestinal homeostasis in the host.

However, it remains unclear whether *Artemisia argyit* essential oil can regulate gut microbiota to strengthen the intestinal barrier and provide therapeutic benefits against *S. pullorum* infection in chicks.

The present study aimed to investigate the effects of *Artemisia argyit* essential oil on the recovery of growth performance and the inhibition of intestinal damage in chicks affected with *S. pullorum*. Additionally, it sought to explore the possible mechanisms behind these effects. This research provides valuable insights into the pathogenesis of *S. pullorum* infection and offers practical guidance for developing targeted interventions.

## Materials and methods

### Bacteria culture conditions

*Salmonella pullorum* (SP)*,* used in this study, was generously provided by Prof. Jingmin Gu from Jilin University. The bacteria were resuscitated in Luria–Bertani (LB, HopeBiol, China) broth culture medium at 37 ℃ and shaken at 200 rpm for 16 h.

*L. reuteri*, also used in this study, was kindly provided by Prof. Yunhe Fu from Jilin University. The bacteria were cultured in MRS (De Man, Rogosa and Sharpe) medium (Qingdao HopeBiol, China) for 24 h under anaerobic conditions at 37 °C.

### Animals and experimental design

Animal experimentation protocols for this study received approval from the Ethics Committees of the Laboratory Animal Centre of Jilin University (No. SY202405028). White-feathered chicks were obtained from the Zhengda Group Breeding Base (Dewei, China). All chicks were randomly assigned in a completely randomised design and were exposed to 16 h of light each day. The room temperature was maintained at 34 °C during the first week and was gradually decreased to 30 °C by the 14^th^ day. All chicks had free access to food and water [30]. The basal diet used in this experiment was formulated in accordance with the nutritional requirements outlined in the Chinese Feeding Standard for Chickens (NY/T33-2004) (Table 1). The animal experiments were conducted in the following three stages.

**Table 1:** **The composition and nutrient levels of diets**

Composition	Percentage (%)
Corn	54.2
Soybean meal	34
Salt	0.3
Dicalcium phosphate	1.5
Limestone	1
Premix^1^	1
Fat-soybean oil	3
Corn gluten	5
Total	100

^1^ Vitamin premix supplied per Kg of diet: Vit A, 12,000 IU; Vit D_3_, 2,500 IU; Vit E, 20 mg; Vit K_3_, 2 mg; Vit B_1_, 2 mg; Vit B_2_, 5 mg; Vit B_6_, 2 mg; Vit B_12,_ 50 mg; niacin, 20 mg; pantothenic acid, 10 mg; folic acid, 1 mg; biotin, 50 mg.

#### *S*.* pullorum* infections in chicks and the therapeutic effects of *Artemisia argyit* essential oil

**Figure 1:**
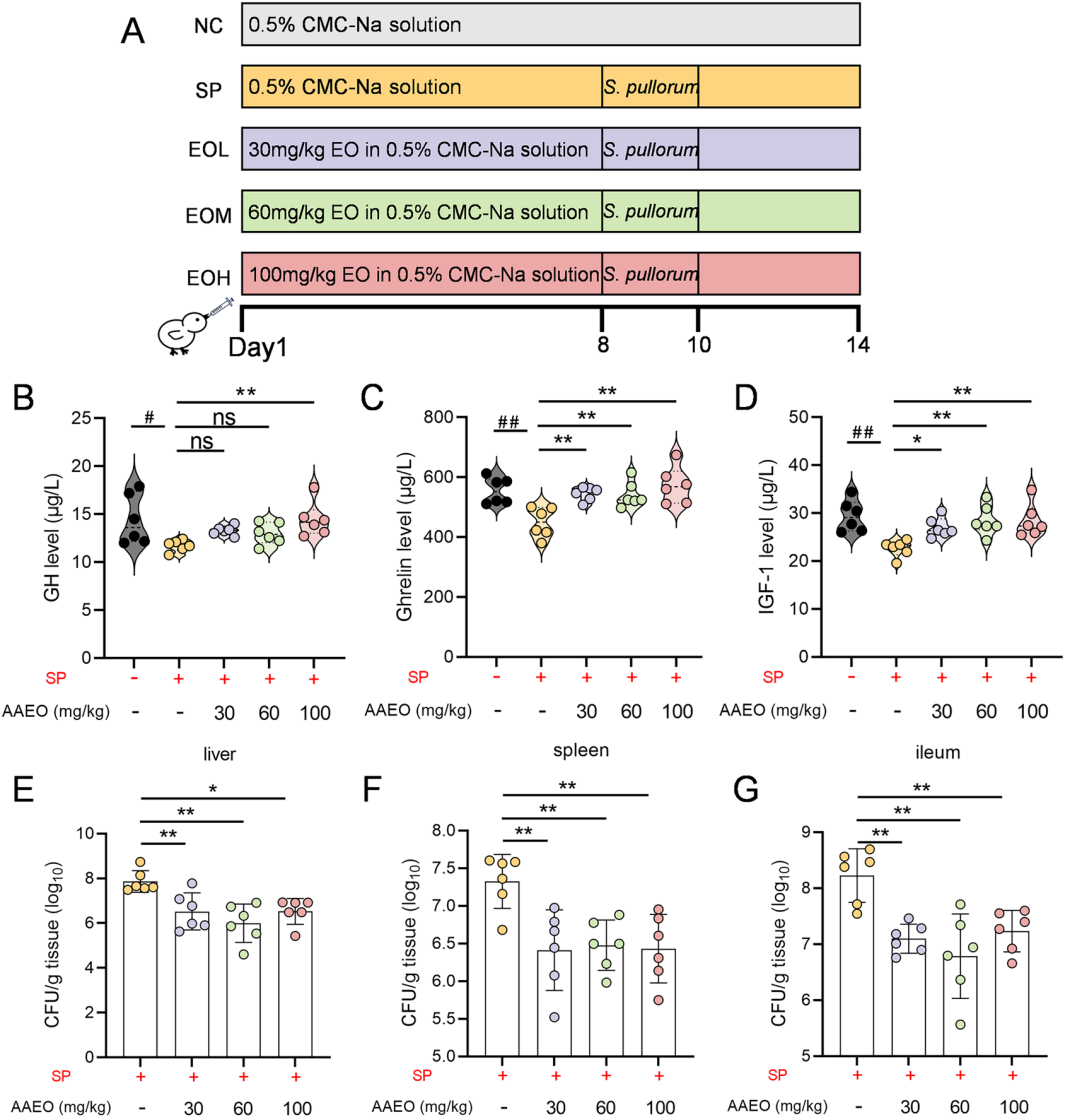
**Effect of *Artemisia argyi* essential oil on growth performance and internal organs bacteria colonization of chicks challenged by *S. pullorum*. A** Experimental protocol of the *Artemisia argyi* essential oil treatment. Chicks were treated with *Artemisia argyi* essential oil for 14 days, and challenged with *S. pullorum* on days 8–10. **B**–**D** Effect of *Artemisia argyi* essential oil on GH (**B**), Ghrelin (**C**), and IGF-1(**D**) levels in serum challenged with *S. pullorum* (*n* = 6). **E–G** Evaluation of *S. pullorum* colonised in the liver (**E**), spleen (**F**) and ileum (**G**) of chicks (*n* = 6). Data are expressed as the mean ± SD. ^#^*P* < 0.05, ^##^*P* < 0.01 vs the uninfected (NC) group; ns *P* > 0.05, *P < 0.05, **P < 0.01 vs the *S. pullorum* infection (SP) group.

Studies have demonstrated the negative effects of *Salmonella* challenges on chick growth performance [31–33]. To assess the protective effect of *Artemisia argyit* essential oil against *S. pullorum*-infections, a model using *S. pullorum*-infected chicks was established (Figure 1A). A total of 150 one-day-old chicks were randomly divided into five treatment groups, with five replicates of six chicks each:

- NC group: chicks challenged with carboxymethyl cellulose (CMC-Na) solution
- SP group: chicks challenged with *S. pullorum*
- EOL, EOM and EOH groups: received 30, 60, and 100 mg/kg/day of *Artemisia argyit* essential oil via oral gavage from day 0 to day 14, respectively.

To ensure consistent and standardised infection, all inoculation experiments involved the oral administration of 500 μL of *S. pullorum* (4 × 10^8^ CFUs/mL) on the 8th day [34], followed by inoculations for three consecutive days. The average daily gain (ADG), average feed intake (ADFI), and feed-to-gain ratio (F/G) were calculated on days 7 and 14 of the study, respectively. On the 14th day, caecal contents were collected and immediately transferred to liquid nitrogen for storage until microbial 16S rRNA analysis.

### Faecal Microbiota Transplantation (FMT)

The faecal suspension was prepared as described previously [35, 36]. Fresh feces from chicks receiving 100 mg/kg/day of *Artemisia argyit* essential oil group for seven days were collected and mixed with sterile phosphate buffered saline (PBS) at a ratio of 1:6. This mixture was then placed in a tissue grinder (JingXin, China) and thoroughly ground. The supernatant was collected and stored for subsequent FMT experiments.

A total of 90 one-day-old chicks were selected as recipients for the FMT treatment and were randomly assigned into three groups: NC, SP and FMT. Each group consisted of five replicates, with six chicks per replicate. The chicks in the FMT group were orally administered 1 mL of the prepared faecal microbiota suspension daily for a consistent 14-day period. During the experiment, the body weights, ADG, ADFI, and F/G of the chicks were recorded on days 7 and day 14. The infection of *S. pullorum* was induced through oral gavage, following the method described previously [34].

### Probiotic validation experiment

One-day-old chicks were randomly divided into three groups (*n* = 6 per group): the NC group, the SP group, and the SP + *L. reuteri* group. The SP + *L. reuteri* group received an administration of *L. reuteri* (1 × 10^8^ CFUs/day/chick) to help re-establish the gut microbiota, before inducing the *S. pullorum* infection model [22]. The *S. pullorum* infection was conducted via oral gavage, following previously established methods [34].

### Samples collection

In therapeutic experiments involving *Artemisia argyit* essential oil and FMT procedures, blood samples were collected from the wing veins on day 14, which marked the end of the experiment. Following this, euthanasia was performed on all chicks via jugular vein puncture. Under aseptic conditions, liver, spleen, and ileum tissues were collected from the subjects. All samples were immediately stored at − 80 °C until analysis. Additionally, ileum samples (approximately 1 cm) were collected and fixed in 4% paraformaldehyde.

### Determination of growth factors in serum

ELISA kits (Jiangsu Meimian Industrial Co., Ltd., China) were used to measure levels of ghrelin, insulin-like growth factor-1 (IGF-1), and growth hormone (GH) serum samples. The serum naturally solidifies at room temperature, and the supernatant was collected by centrifugation at 12 000 rpm for 10 min.

### Determination of bacteria in the internal organs

The bacterial load in *Salmonella*-infected chicks was enumerated following previously described methods [37]. Liver, spleen, and ileum samples were weighed and then homogenised in sterile PBS for 30 min. The homogenate was diluted to the appropriate volume with sterile PBS, and each sample was placed on Xylose Lysine Deoxycholate (XLD) agar plates for microorganism counting.

### Histological analysis

Chick ileum tissue was fixed in 4% paraformaldehyde, embedded in paraffin, and stained with haematoxylin and eosin (H&E) [38]. The intestinal morphology of various groups was observed using light microscopy. Villus height and crypt depth were measured using CaseViewer software, and the ratio of villus height to crypt depth (V/C) was calculated.

### Total RNA extraction and quantitative RT-qPCR

As previously described [39], RNA was extracted from frozen ileum tissues using TransZol Up (TransGen, China). The extracted RNA was then reverse transcribed using a cDNA reverse transcription kit (Mei5bio, China). Real-time qPCR was conducted on a Step One Plus system (Applied Biosystems, Foster City, USA). β-actin served as the internal control, and the 2^−△△Ct^ method was utilised to quantify the relative mRNA expression levels of the genes [40]. The primers used in this analysis are detailed in Additional File 1 [41].

### Inflammatory cytokine and oxidative stress factor detection

ELISA kits (Jiangsu Meimian Industrial Co., Ltd., China) were used to measure the levels of key inflammatory cytokines, including interleukin-6 (IL-6), interleukin-10 (IL-10), interleukin-1β (IL-1β), and nuclear factor kappa-B (NF-κB), as well as oxidative stress factors such as malondialdehyde (MDA), superoxide dismutase (SOD) in ileum tissue samples. To prepare a homogenate of the ileum tissue, PBS was used, and the supernatant was collected by centrifugation at 12 000 rpm for 10 min at 4 °C. The levels of corresponding cytokines were detected following the manufacturer's instructions, and absorbance was measured at 450 nm using a microplate reader (Tecan, CH).

### 16S ribosomal RNA (16S rRNA) sequencing and data analysis

Microbial DNA was extracted from the caecal contents of chicks (TianGen, China) following the manufacturer’s protocol. Spike-in sequences, along with bacterial 16S rRNA sequences from each DNA sample, were amplified across the variable regions V3-V4 using primers F (5′-GTGCCAGCMGCCGCGGTAA-3′) and R (5′-GGACTACHVGGGTWTCTAAT-3′). The amplifies sequences were then sequenced by Novogene Inc. (China) on the Novaseq 6000 platform (Illumina, San Diego, USA).

The raw data obtained from the sequencing process contained some interference data, which could affect the accuracy of the information analysis. To address this, the raw data was aggregated and filtered to obtain valid data. Noise reduction was then performed using DADA2 or denoising techniques, resulting in the final ASVs.

### Statistical analysis

GraphPad Prism 9.3 (GraphPad software) was utilised for statistical analysis. All data are presented as the mean ± SD. Significant differences between the two groups were assessed using a *t*-test (parametric). For comparisons involving more than two groups, one-way ANOVA was employed. The *P* values are categorised as follows: ns *P* > 0.05; *P < 0.05; **P < 0.01.

For analysing caecal microbiota data, a *t*-test and Wilcoxom rank-sum test were applied for two subgroups, while Tukey and Kruskal–Wallis rank-sum tests were used for groups with more than two subgroups to calculate alpha diversity. The significance of segregation in PCoA was tested using R software version 4.0.3 with a weighted UniFrac distance measure. Linear discriminant analysis (LDA) combined with effect size measurements (LEfSe) was used to identify bacterial differences among groups. The functional contents of the intestinal metagenome was estimated using PICRUSt 2.

## Results

### *Artemisia argyit* essential oil improves the health of chicks affected by *S. pullorum* infection

The growth performance of the chicks at two stages (days 1 to 7 and days 7 to 14) was presented in Table 2. From days 1 to 7, before the *S. pullorum* challenge, *Artemisia argyit* essential oil supplementation (30, 60, and 100 mg/kg) exerted no significant effect on BW, ADG, ADFI and F/G of chicks compared with the NC group (*P* > 0.05). However, from day 7 to 14, the results showed that *S. pullorum* infection substantially reduced the BW, ADG, ADFI and potentiated the F/G of chicks in comparison with the NC group (*P* < 0.05). Compared with the infected group, the supplementation of 100 mg/kg *Artemisia argyit* essential oil significantly increased BW, ADG, ADFI and reduced the F/G of chicks (*P* < 0.05). Based on the above findings, *Artemisia argyit* essential oil addition alleviated growth retardation of *S. pullorum*-challenged chicks.

**Table 2:** **Effect of *Artemisia argyit* essential oil on growth performance of chicks challenged by *S. pullorum***

Time	Items	Groups					*P* value
		**NC**	***SP***	**EOL**	**EOM**	**EOH**	
d7	BW g/chick	88.65 ± 2.36^a^	87.73 ± 2.25^a^	87.98 ± 2.45^a^	87.53 ± 2.44^a^	87.75 ± 2.69^a^	0.44
d14		160.90 ± 2.75^b^	129.25 ± 3.10^a^	153.25 ± 4.39^bc^	165.75 ± 8.87^bc^	155.78 ± 4.75^bc^	< 0.05
d1-7	ADG g/chick/d	9.81 ± 0.34^a^	9.68 ± 0.32^a^	9.71 ± 0.35^a^	9.65 ± 0.35^a^	9.68 ± 0.38^a^	0.44
d7-14		10.32 ± 0.51^b^	5.93 ± 0.57^a^	9.32 ± 0.69^bc^	11.17 ± 1.32^bc^	9.72 ± 0.93^b^	< 0.05
d1-7	ADFI g/chick/d	15.73 ± 1.77^a^	15.76 ± 2.15^a^	15.65 ± 2.12^a^	15.82 ± 1.77^a^	15.56 ± 1.87^a^	0.08
d7-14		17.38 ± 2.00^b^	11.00 ± 2.02^a^	16.72 ± 1.87^b^	18.34 ± 1.64^b^	16.19 ± 2.06^b^	< 0.05
d1-7	F/G	1.61 ± 0.19^a^	1.63 ± 0.21^a^	1.61 ± 0.23^a^	1.64 ± 0.17^a^	1.61 ± 0.20^a^	0.96
d7-14		1.69 ± 0.21^b^	1.87 ± 0.35^a^	1.80 ± 0.24^a^	1.66 ± 0.22^b^	1.68 ± 0.28^b^	< 0.05

BW body weight, ADG average daily gain, ADFI average daily feed intake, F/G feed conversion

NC negative control, *SP* positive control (chicks were challenged with *S. pullorum* from 8 to 10 days of age); EOL, EOM, EOH chicks supplemented with 30 mg/kg, 60 mg/kg, 100 mg/kg *Artemisia argyit* essential oil, respectively.

^a–c^Means within a row with different superscripts are significantly different (*P* < 0.05). *P*-value represent the difference comparison between group NC, SP, EOL, EOM and EOH groups.

Since the essential oil of *Artemisia argyit* improved chick growth performance, we further analysed whether the growth-related hormones in chick serum were affected. We found that the levels of GH, Ghrelin, and IGF-1 secretion were significantly lower in the *S. pullorum*-infected group compared to the NC group. In contrast, the levels of Ghrelin and IGF-1 were dose-dependently elevated in chicks fed *Artemisia argyit* essential oil (30, 60, and 100 mg/kg) when compared to the SP group (*P* < 0.05). Notably, only the chicks supplemented with 100 mg/kg of *Artemisia argyit* essential oil showed increased levels of GH (*P* < 0.05, Figure 1B–D).

Next, we examined the effects of the 100 mg/kg supplementation of *Artemisia argyit* essential oil on chick physiology and gut microbiota in the following analyses.

Additionally, the supplementation of *Artemisia argyit* essential oil reduced the burden of *S. pullorum* in the liver, spleen, and ileum of chicks compared to the *S. pullorum-*infected group (*P* < 0.05, Figure 1E–G). Overall, these results suggested that *Artemisia argyit* essential oil may help alleviate *S. pullorum*-infection in chicks by reducing bacterial colonisation and preventing translocation of bacteria.

### *Artemisia argyit* essential oil inhibits damage caused by *S. pullorum* and prevents the excessive release of inflammatory factors

**Figure 2:**
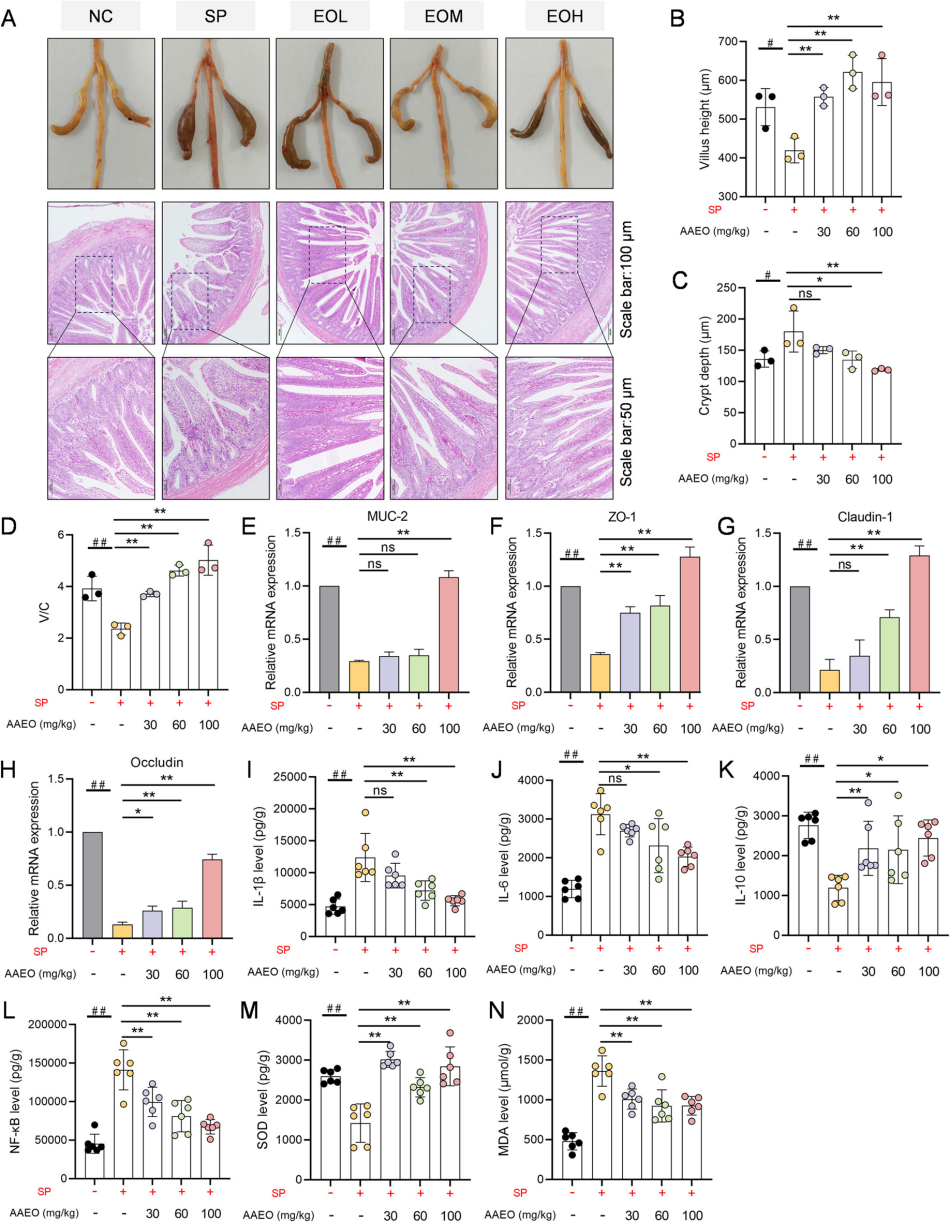
**Protective effect of *Artemisia argyi* essential oil on intestinal damage caused by *S. pullorum* infection. A** Intestinal lesions and H&E staining in *S. pullorum*-infected chicks treated with or without *Artemisia argyi* essential oil. **B**–**D** The villus height (**B**), crypt length (**C**), and ratio of the villus to crypt length (**D**) of the ileum (*n* = 3). **E–H** Ileum *MUC-2* (**E**), *ZO-1* (**F**), *Claudin-1* (**G**), and *Occludin* (**H**) mRNA levels were assessed by qPCR in chicks (*n* = 3). **I**–**N** Evaluation of chick ileum IL-1β (**I**), IL-6 (**J**), IL-10 (**K**), NF-κB (**L**), SOD (**M**), and MDA (**N**) levels with ELISA kits (*n* = 6). Data are expressed as the mean ± SD. ^#^*P* < 0.05, ^##^*P* < 0.01 vs the uninfected (NC) group; ns *P* > 0.05, *P < 0.05, **P < 0.01 vs the *S. pullorum* infection (SP) group.

Due to the negative effects of *S. pullorum* invading intestinal cells, we investigated the protective effect of *Artemisia argyit* essential oil on the intestines of chicks infected with *S. pullorum*. As illustrated in Figure 2A, the intestines of chicks infected with *S. pullorum* exhibited swelling of the ileum and significant accumulation of digestive products in the caecum. This condition was alleviated following treatment with *Artemisia argyit* essential oil. Specifically, the ileum crypt depth in the chicks decreased (*P* < 0.05), while both villus height and the V/C were significantly improved (*P* < 0.05) after treatment with *Artemisia argyit* essential oil (Figure 2B–D).

Moreover, the expression of mucosa barrier-related genes in the ileum revealed that *S. pullorum* infection downregulated the expression of *ZO-1, Claudin-1*, *MUC-2*, and *Occludin* (*P* < 0.05), which was reversed by supplementation with 100 mg/kg of *Artemisia argyit* essential oil (*P* < 0.05, Figure 2E–H). This suggested that *Artemisia argyit* essential oil may help restore intestinal barrier integrity.

In terms of inflammatory cytokines in the ileum, the infiltration of pro-inflammatory factor IL-1β, IL-6, and NF-κB cytokines significantly increased in the ileum tissues of chicks infected with *S. pullorum* (*P* < 0.05). Conversely, the anti-inflammatory factor IL-10 was notably reduced due to the infection (*P* < 0.05). Notably, the antioxidant defense functions in chicks treated with *S. pullorum* were impaired, as indicated by a reduction in the antioxidant enzyme SOD and an increase in the peroxidation product MDA levels (*P* < 0.05).

However, treatment with *Artemisia argyit* essential oil mitigated the *S. pullorum*-induced inflammatory markers (*P* < 0.05, Figure 2I–L) and improved antioxidant function (*P* < 0.05, Figure 2M, N). Collectively, these results suggested that *Artemisia argyit* essential oil is effective in alleviating elevated intestinal inflammatory factors and restores intestinal barrier function in chicks infected with *S. pullorum*.

### *Artemisia argyit* essential oil alters the structure of the gut microbial community in chick

**Figure 3:**
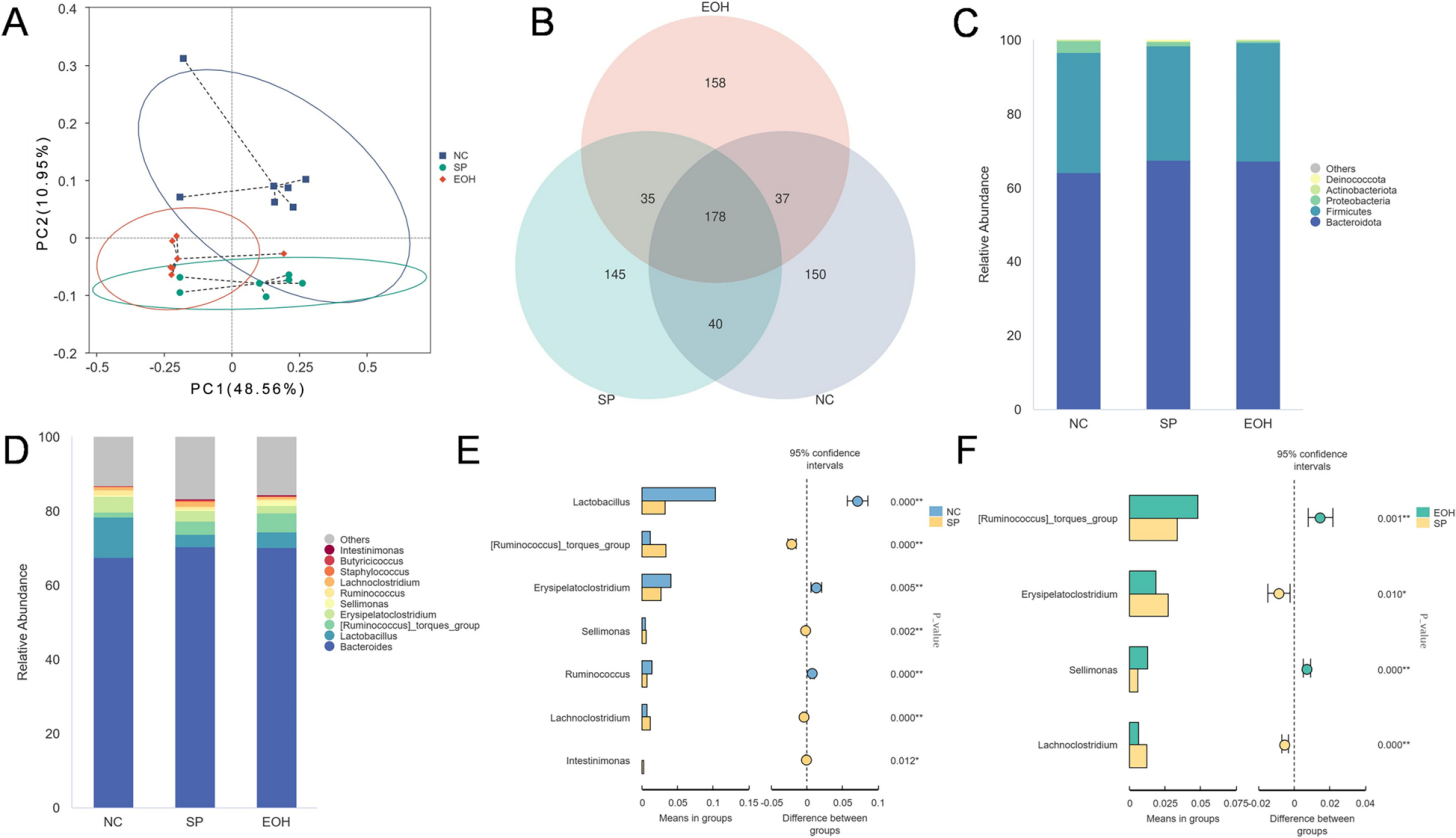
**Microbiome analysis of faecal samples from *S. pullorum*-infected chicks treated with or without *Artemisia argyi* essential oil. A** PCoA analysis of the gut microbiota of healthy (NC), *S. pullorum*-infected (SP) and chicks treated with 100 mg/kg/day of *Artemisia argyi* essential oil and SP infection chicks (EOH), data were analysed using unweighted UniFrac (*n* = 6). **B** Comparative analysis of gut microbial OTUs of NC, SP, and EOH group chicks (*n* = 6). **C** The top 5 species at the phylum levels among different groups (*n* = 6). **D** The top 10 species at the genus levels among different groups (*n* = 6). **E**, **F** Differential caecal microbiota at genus levels among different groups (*n* = 6). NC: Uninfected chicks, SP: Chicks infected with *S. pullorum* (SP), EOH: Supplementation of 100 mg/kg/day *Artemisia argyi* essential oil (EOH) and *S. pullorum*-infected chicks.

To investigate the effects of *Artemisia argyit* essential oil on the gut microbiota of chicks, we conducted 16S rRNA sequencing on cecal content samples from chicks subjected to the different treatments. There were no significant differences in the alpha-diversity indicators of gut microbiota among the groups (*P* > 0.05, Additional file 2). To analyse the similarity or differences in gut microbial community structure across samples, we assessed the β-diversity of cecal microorganisms using PCoA analysis. As shown in Figure 3A, the PCoA analysis revealed a significant difference in the composition of gut microbiota among the three groups.

In this study, we identified 743 OTUs in the cecal contents of all groups, with 178 core OTUs shared among all groups. Additionally, there were 150, 145, and 158 OTUs unique to the NC, SP, and EOH groups, respectively (Figure 3B). Differences in microbial abundance at the phylum level among all groups were illustrated in Figure 3C. The top five dominant phyla identified were Bacteroidota, Firmicutes, Proteobacteria, Actinobacteriota, and Deinococcota.

Figure 3D highlights the major bacterial genera enriched in the cecal contents, with *Bacteroides*, *Lactobacillus*, *Ruminococcus*, *Erysipelatoclostridium,* and *Sellimonas* being the most prominent. Notably, at the genus level, *Ruminococcus* and *Sellimonas* were enriched in the EOH group, compared to the SP group. Conversely, *Lactobacillus* was found to be depleted in the SP group compared to the NC group (Figure 3E and F).

These findings demonstrated that *S. pullorum* infection disturbs the stability and function of the chick gut microbiota. However, significant changes in the composition of gut microbiota were observed following supplementation with *Artemisia argyit* essential oil. Importantly, the dominant gut microbiota in chicks was found to be significantly greater than in the SP group, suggesting that gut microbes may play a beneficial role in the inflammatory response to *S. pullorum* infection.

### FMT of *Artemisia argyit* essential oil donor microbiota protects chicks from *S. pullorum* infection

**Figure 4:**
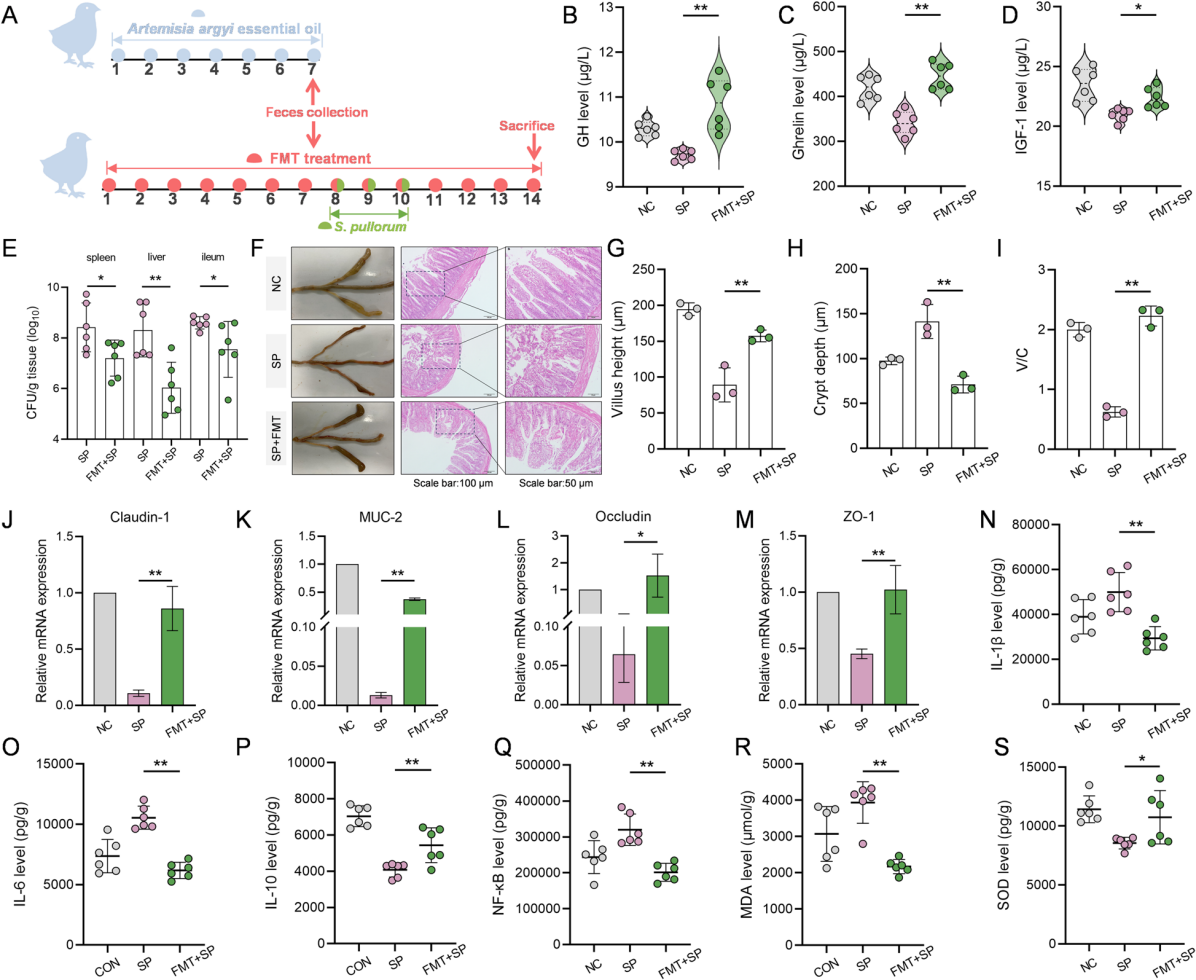
**FMT from *Artemisia argyi* essential oil treated chicks alleviates *S. pullorum* infection in chicks. A** Illustration of FMT. FMT model in *S. pullorum*-infected chicks (NC: chicks without FMT treatment; SP: *S. pullorum*-infected chicks; FMT + SP: supplementation of FMT in *S. pullorum*-infected chicks (*n* = 6). **B**–**D** Effect of FMT on GH (**B**), Ghrelin (**C**), and IGF-1 (**D**) levels in serum of *S. pullorum*-infected chicks (*n* = 6). **E** Evaluation of *S. pullorum* colonization in chick visceral after FMT treatment (*n* = 6). **F**–**I** Intestinal lesions and histopathological (H&E staining) representative images of lesions in FMT treated *S. pullorum*-infected chicks (**F**), the villus height (**G**), crypt length (**H**), V/C (**I**) of the ileum (*n* = 3). **J**–**M** The mRNA levels of chick ileum mucosal genes *Claudin-1* **(J)**, *MUC-2* **(K)**, *Occludin* **(L)** and *ZO-1* (**M**) were assessed by qPCR (*n* = 3). **N**–**S** Inflammatory parameters of ileum from different groups, including IL-1β (**N**), IL-6 (**O**), IL-10 (**P**), NF-κB (**Q**), MDA (**R**) and SOD (**S**) concentrations were measured (*n* = 6). Data are expressed as the mean ± SD. *P < 0.05, **P < 0.01 vs the *S. pullorum* infection (SP) group.

The FMT model was established to investigate the potential of gut microbiota in mediating the protective effects of *Artemisia argyit* essential oil against *S. pullorum* infection in chicks (Figure 4A). Our findings indicated that the FMT group exhibited greater therapeutic benefits compared to the SP group. This was evident through increased levels of growth-related hormones (GH, Ghrelin, and IGF-1) (*P* < 0.05, Figure 4B–D), as well as a reduction in bacterial burden in tissue (*P* < 0.05, Figure 4E).

Additionally, chicks in the FMT group significant increases in body weight, ADG, and ADFI compared to the SP group from days 7 to 14. The F/G ratios for the FMT group were also considerably lower (*P* < 0.05) than those of the SP group (Table 3).

**Table 3:** **Effect of FMT supplementation on growth performance of chicks challenged with** ***S. pullorum***

Time	Items	Groups			*P* value
		NC	SP	FMT	
d7	BW g/chick	87.22 ± 3.31^a^	85.13 ± 4.01^a^	85.29 ± 3.57^a^	0.05
d14		160.60 ± 3.38^b^	131.80 ± 2.88^a^	155.32 ± 4.39^bc^	< 0.05
d1-7	ADG g/chick/d	9.60 ± 0.47^a^	9.30 ± 0.57^a^	9.33 ± 0.51^a^	0.05
d7-14		10.48 ± 0.65^b^	6.67 ± 0.69^a^	10.00 ± 0.80^bc^	< 0.05
d1-7	ADFI g/chick/d	14.45 ± 1.54^a^	14.76 ± 1.59^a^	14.89 ± 1.70^a^	0.56
d7-14		17.05 ± 1.66^b^	13.84 ± 1.43^a^	16.98 ± 2.26^b^	< 0.05
d1-7	F/G	1.51 ± 0.18^a^	1.59 ± 0.17^a^	1.60 ± 0.20^a^	0.11
d7-14		1.63 ± 0.16^b^	1.87 ± 0.06^a^	1.69 ± 0.02^b^	< 0.05

NC negative control, *SP* positive control (chicks were challenged with *S. pullorum* from 8 to 10 days of age); FMT group were orally administered faecal microbiota suspension (fresh faeces from chicks fed continuously with Artemisia essential oil for 7 days).

^a–c^ Means within a row with different superscripts are significantly different (*P* < 0.05). *P*-value represent the difference comparison between group NC, SP and FMT groups.

Moreover, the FMT group exhibited less intestinal injury relative to the SP group, as shown by improved ileum villi height and V/C, along with a reduced crypt depth (*P* < 0.05, Figure 4F–I). Similar to the results observed with *Artemisia argyit* essential oil treatment, FMT corrected the decreases in the ileum TJ proteins *Claudin-1*, *Occludin, ZO-1*, as well as mucin *MUC-2,* which were caused by *S. pullorum* infection in chicks (*P* < 0.05, Figure 4J–M).

Consistently, FMT lowered the concentrations ileal cytokines, such as IL-1β, IL-6, NF-κB, and the peroxidation product MDA, while increasing levels of the anti-inflammatory factor IL-10 and the antioxidant enzyme SOD compared to the *S. pullorum* infection (*P* < 0.05, Figure 4N–S). Overall, FMT enhanced resistance to *S. pullorum* infection in chicks, suggesting that the effectiveness of *Artemisia argyit* essential oil against *S. pullorum* infection is mediated by the modulation of gut microbiota.

### Oral administration of *Artemisia argyit* essential oil elevates the accumulation of probiotics in the gut microbiota

To investigate the interaction between *Artemisia argyit* essential oil, gut microbiota, and *S. pullorum* infection, we collected the caecal contents of chicks that were supplemented with *Artemisia argyit* essential oil for seven consecutive days. These samples underwent 16S rRNA sequencing to assess the effectiveness of the treatment in improving the gut microbiota of chicks.

**Figure 5:**
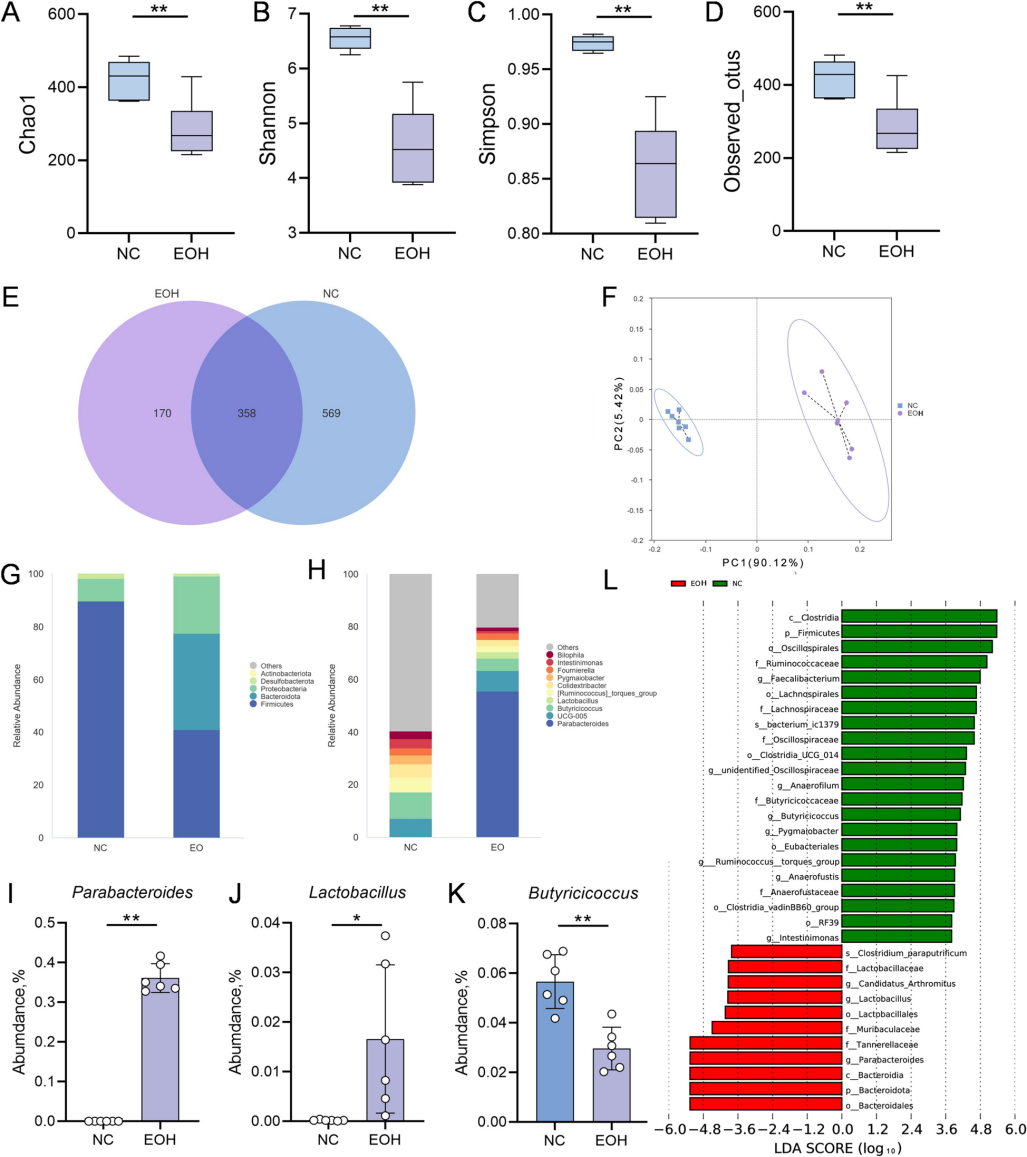
**Effects of *Artemisia argyi* essential oil on the microbial communities in the intestinal of recipient chicks.** One-day-old chick were given 100 mg/kg/day of *Artemisia argyi* essential oil continuously for 7 days, and faecal microbiota was evaluated using 16S rRNA sequencing on day 7. **A**–**D** Chao1 index (**A**), Shannon index (**B**), Simpson index (**C**), and Observed_otus (**D**) showed that EOH group has reduced alpha-diversity, compared to the NC group (*n* = 6). **E** Vene analyses demonstrated different OTUs in the NC group and *Artemisia argyi* essential oil treatment group (EOH) (*n* = 6). **F** PCoA scores of chicks fecal based on unweighted unifrac scores (*n* = 6), indicated that the composition of the gut microbiota was different in the two groups. **G**–**H** Bacterial composition of the indicated groups at the phylum (**G**) and genus (**H**) levels (*n* = 6). **I**–**K** The relative abundance of *Parabacteroides* (**I**), *Lactobacillus* (**J**), and *Butyricoccus* (**K**) at genus levels (*n* = 6). **L** LEfSe was performed to show several bacterial taxa enriched in NC and EOH groups (log_10_LDA score > 4). NC: Uninfected chick group, EOH: Continuous feeding of 100 mg/kg/day of *Artemisia argyi* essential oil for 7 days. Data are expressed as the mean ± SD. **P < 0.01 vs the NC group.

Alpha-diversity indices—specifically Chao1, Shannon, Simpson, and Observed OTUs—were significantly lower in the EOH group compared to the NC group (Figure 5A–D, *P* < 0.05). Venn analysis indicated that the gut microbiota of chicks treated with *Artemisia argyit* essential oil showed marked improvements compared to the NC group. The EOH group exhibited a reduction in observed operational taxonomic units (OTUs) compared to the NC group (Figure 5E). Consistently, PCoA evaluation revealed a significant separation in gut microbial structure between the two groups (Figure 5F).

Subsequent calculations of the relative abundances of bacteria following species annotation (Figure 5G–H) showed that the abundances of *Lactobacillus* and *Parabacteroides* increased after treatment with *Artemisia argyit* essential oil, while the abundance of *Butyricicoccus* decreased (Figure 5I–K, P < 0.05). Additionally, a heatmap (Additional file 3A), and LEfSe (Figure 5L) were employed to analyse species differences. The results of these comparisons were consistent, with *Lactobacillus* being significantly after the treatment.

Furthermore, functional annotation analysis based on the KEGG database indicated that after treatment with *Artemisia argyit* essential oil, there was an increased enrichment of genes related to glycosyltransferase involved in cell wall biosynthesis pathways (Additional file 3B). These findings suggested that *Artemisia argyit* essential oil can remodel gut microbiota, potentially providing protection against *S. pullorum* infection.

### Commensal *L. reuteri *alleviates* S. pullorum* infection-aggravated intestinal damage in chicks

According to the 16S rRNA sequencing analysis presented in Figure 5, the primary differential probiotics among gut microbes in chicks treated with *Artemisia argyit* essential oil were *Lactobacillus* and *Parabacteroides*. Notably, while *Parabacteroides* is considered a core member of the gut microbiota, it has been identified as a producer of LPS [42].

**Figure 6:**
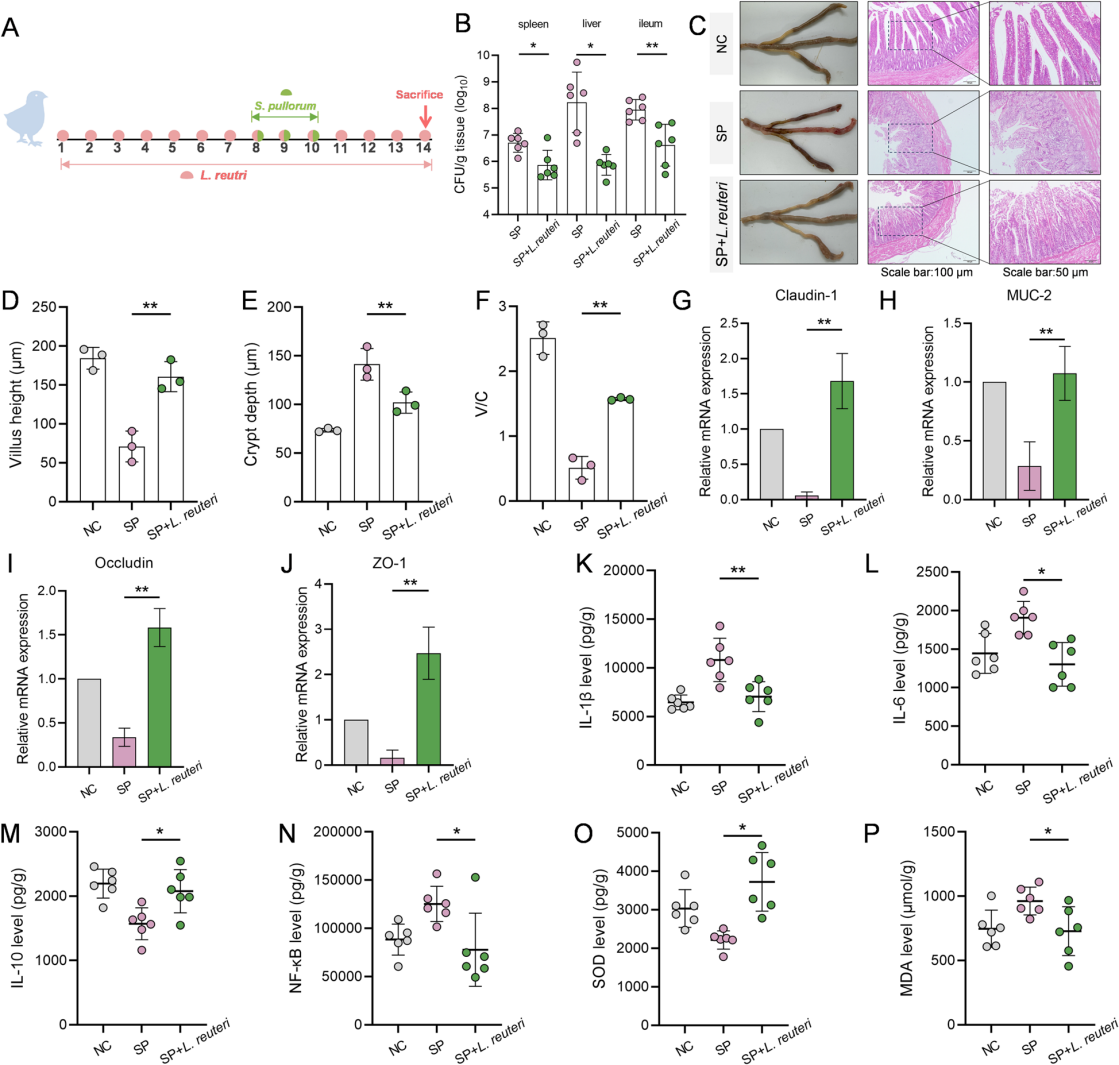
**Effects of supplementation with *L. reuteri* against *S. pullorum*-infected chicks. A** Schematic diagram of *L. reuteri* fed to treat *S. pullorum*-infected chicks. Chicks were treated with *L. reuteri* for 14 days, and challenged with *S. pullorum* on days 8–10. **B** Protective effect of *L. reuteri* feeding against bacterial colonization of the visceral of *S. pullorum*-infected chicks (n = 6). **C** Effects of *L. reuteri* supplementation on the morphological structure of the ileum intestine of *S. pullorum*-infected chicks. **D**–**F** The villus height (**D**), crypt length (**E**), and ratio of the villus to crypt length (**F**) of the ileum (*n* = 3). **G**–**J** Gene expression levels of *Claudin-1* (**G**), *MUC-2* (**H**), *Occludin* (**I**), and *ZO-1* (**J**) in chick ileum were determined by qPCR, and normalised by *β-actin* expression (*n* = 3). **K**–**P** Determination of inflammatory factor IL-1β (**K**), IL-6 (**L**), IL-10 (**M**), NF-κB (**N**) and SOD (**O**), MDA (**P**) levels in chick ileum by ELISA (*n* = 6). Data are expressed as the mean ± SD. *P < 0.05, **P < 0.01 vs the *S. pullorum* infection (SP) group.

Furthermore, we investigated the intrinsic mechanisms by which *L. reuteri* may restore the dysfunction of the intestinal epithelial barrier caused by *S. pullorum* infection. We developed an *L. reuteri* protection model to assess its effects on the *S. pullorum* infection process in chicks (Figure 6A). As anticipated, the presence of *L. reuteri* reduced both the invasion of bacteria into internal organs (Figure 6B) and intestinal damage (Figure 6C). We observed a significant increase in the length of the ileal villi in chicks from the *L. reuteri* group (*P* < 0.05), as well as a substantially higher villus-to-crypt length ratio (*P* < 0.05) compared to the SP group (Figure 6D–F).

Additionally, we measured indicators related to barrier integrity and inflammation in the ileum. As depicted in Figure 6G–J, the expression levels of *ZO-1*, *MUC-2*, *Claudin-1,* and *Occludin* mRNA were significantly higher in the ileum of the *L. reuteri* group (*P* < 0.05). Correspondingly, the Elisa results indicated that the *L. reuteri* intervention group had significantly lower levels of IL-1β, IL-6, NF-κB, and the overoxidation product MDA compared to the SP group. In contrast, the levels of IL-10 and the antioxidant enzyme SOD was markedly elevated in the *L. reuteri* intervention group (*P* < 0.05, Figure 6K–P).

Collectively, these findings suggested that the ingestion of *L. reuteri* may provide beneficial physiological effects to mitigate *S. pullorum* infection in chicks.

### *Artemisia argyit* essential oil activates the Wnt/β-catenin pathway to repair *S. pullorum*-induced intestinal epithelia damage

**Figure 7:**
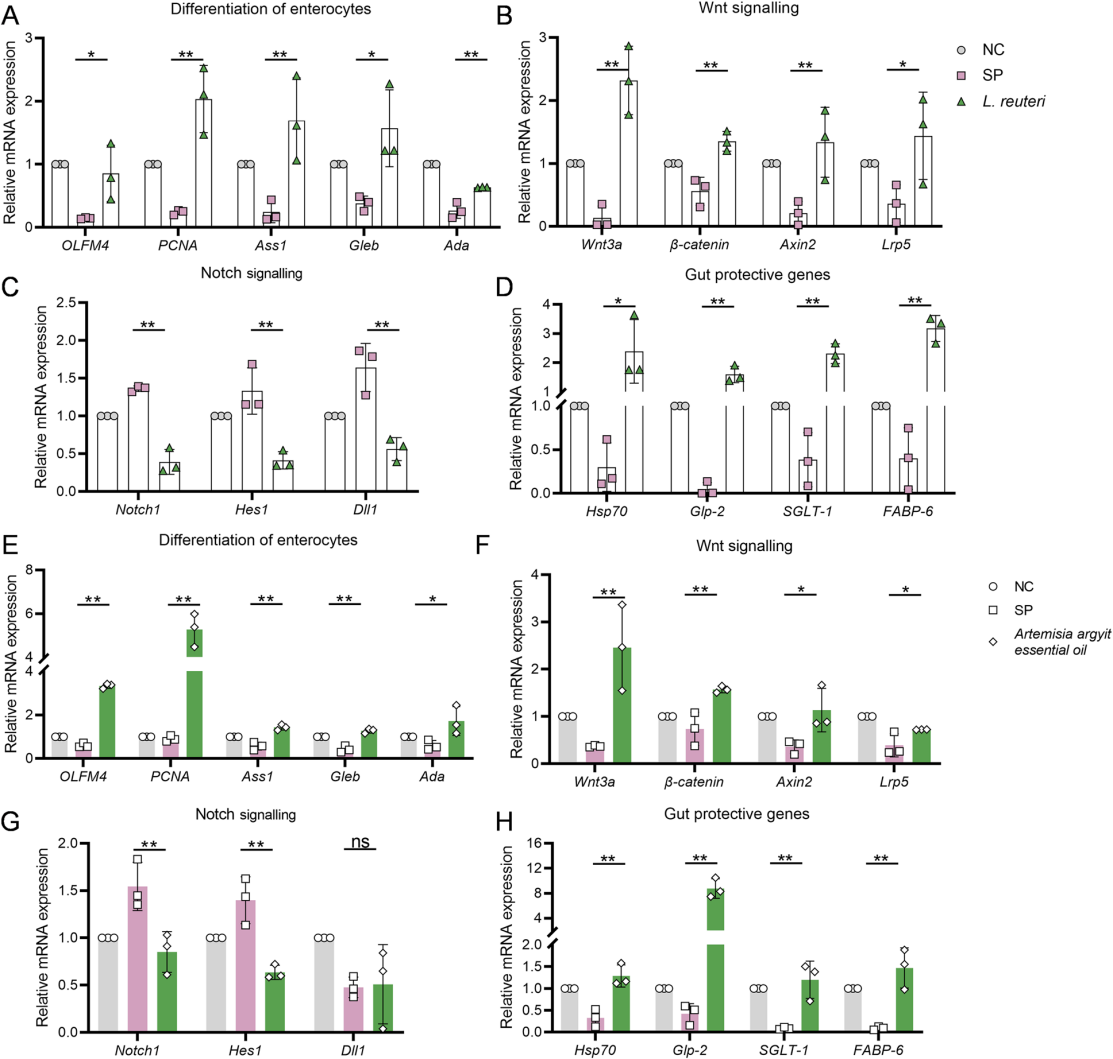
**Supplementation with *Artemisia argyi* essential oil or *L. reuteri* activates the Wnt/β-catenin signalling pathway in *S. pullorum*-infected chicks. A** and **E** Supplementation with *L. reuteri* (**A**) or *Artemisia argyi* essential oil (**E**) upregulated mRNA expressions of genes associated with intestinal epithelial differentiation during *S. pullorum* infection (*n* = 3). **B** and **F** The comparison of Wnt/β-catenin signalling genes expression in the ileum of *L. reuteri* (**B**) or *Artemisia argyi* essential oil (**F**) treated *S. pullorum*-infected chicks (*n* = 3). **C** and **G** The expression levels of Notch signalling genes in the ileum of *S. pullorum*-infected chicks treated with *L. reuteri* (**C**) or *Artemisia argyi* essential oil (**G**) were measured by qPCR (*n* = 3). **D** and **H*** L. reuteri* (**D**) or *Artemisia argyi* essential oil (**H**) treatment increased the expression of intestinal protective genes in the ileum of *S. pullorum*-infected chicks (*n* = 3). Data are expressed as the mean ± SD. ns *P* > 0.05, *P < 0.05, **P < 0.01 vs the *S. pullorum* infection (SP) group.

Since higher resistance to *S. pullorum* and lower bacterial burdens were observed in the groups treated with *Artemisia argyit* essential oil and *L. reuteri*, we further examined the protective mechanisms of these treatments against *S. pullorum* infection. The alteration in the intestinal barrier and the structure of the intestinal mucosa are closely linked to the differentiation of enterocytes. As expected, several intestinal cell differentiation genes—including olfactomedin 4 (*OLFM4*), proliferating cell nuclear antigen (*PCNA*), arginosuccinate synthetase 1 (*Ass1*), and β-galactosidase (*Gleb*), and adenosine deaminase (*Ada*)—were upregulated in the *L. reuteri* treatment group compared to the SP group (*P* < 0.05, Figure 7A). This upregulation indicates that *L. reuteri* positively influences the development of intestinal epithelial cells. The observed increase in epithelial proliferation may be attributed to the activation of the Wnt/β-catenin signalling pathway and the inhibition of the Notch pathway. As shown in Figure 7B, the *L. reuteri* group significantly increased mRNA expression of *Wnt3a*, *β-catenin*, *Axin2,* and *Lrp5*, indicating activation of the Wnt/β-catenin signalling pathway (*P* < 0.05). Additionally, the *L. reuteri* group exhibited a decreased expression of Notch signalling pathway genes (*Dll1*, *Notch1*, and *Hes1*) (*P* < 0.05, Figure 7C). Finally, we also investigated several promising gut-protecting genes. The results showed that *L. reuteri* treatment substantially upregulated the expression of *HSP70*, sodium-dependent glucose transporter 1 (*SGLT-1*), fatty acid-binding protein 6 (*FABP-6*), and glucagon-like peptide 2 (*GLP-2*), compared to the SP group (*P* < 0.05, Figure 7D).

Considering that *Artemisia argyit* essential oil may mediate the enrichment of gut microbes, particularly *Lactobacillus*, we investigated whether this oil activated the Wnt/β-catenin pathway. Notably, *Artemisia argyit* essential oil enhanced the mRNA expression of enterocyte differentiation markers such as *OLFM4*. Additionally, it significantly increased the expression levels of *PCNA*, *Ass1*, *Gleb*, and *Ada* (*P* < 0.05, Figure 7E). These findings suggested that *Artemisia argyit* essential oil promotes the proliferation and differentiation of intestinal epithelial cells.

Compared to the *S. pullorum* infection group, the treatment with *Artemisia argyit* essential oil markedly upregulated the expression of *Wnt3a*, *β-catenin*, *Axin2*, and *Lrp5*. This was consistent with the enhanced expression observed with *L. reuteri* treatments, indicating that the Wnt/β-catenin signalling pathway was activated by *Artemisia argyit* essential oil (*P* < 0.05, Figure 7F). In contrast, Paneth cells play a crucial role in the proliferation of intestinal stem cells (ISCs) through the secretion of the Notch ligand Dll [43]. However, *Artemisia argyit* essential oil showed a decreasing trend in the expression of Notch signalling pathway genes (*Notch1* and *Hes1*), suggesting that the Notch signalling pathway was effectively inhibited by this essential oil (*P* < 0.05, Figure 7G).

Furthermore, *Artemisia argyit* essential oil promoted the expression of intestinal protective genes, including *HSP70*, *Glp-2*, *SGLT-1*, and *FABP-6* (*P* < 0.05, Figure 7H). These results indicated that *L. reuteri*, produced in the presence of *Artemisia argyit* essential oil, mediates the activation of the Wnt/β-catenin pathway and mitigates the intestinal damage induced by *S. pullorum* in chicks.

## Discussion

Pullorum disease, caused by *S. pullorum,* poses a significant global health challenge for livestock, leading to reduced production performance, intestinal colonisation, and invasion of internal organs in chicks [44, 45]. Notably, chicks are highly susceptible to *S. pullorum* infection during their first 14 days of life due to their developing gut microbiota [46]. Traditionally, antibiotics have been used to manage *Salmonella* infections; however, the rapid emergence of multidrug-resistant (MDR) *Salmonella* strains presents a serious threat to the poultry industry and public health [47]. Consequently, reducing antibiotic has become a crucial necessity. Since 2020, China has implemented strict policies to limit and reduce antibiotic usage in agriculture [48].

In light of this, essential oils are being considered as suitable candidates due to their safety and potential health benefits [49]. This study specifically investigates the effects of *Artemisia argyit* essential oil on preventing and treating *S. pullorum* infections by examining its impact on gut microbiota. Our research provides evidence that treatment with *Artemisia argyit* essential oil alleviates intestinal mucosal barrier damage and intestinal inflammation caused by *S. pullorum* infection. This is achieved through the remodelling of gut microbiota, particularly through the promotion of *L. reuteri*. These findings offer a new perspective on regulating intestinal health for the treatment of *S. pullorum* infections.

This further study investigated the effectiveness of *Artemisia argyit* essential oil in reducing the inflammatory response and restoring the integrity of the intestinal mucosal barrier in chicks infected with *S. pullorum*. Changes in chick weight may correlate with intestinal barrier integrity. Research shows that *Salmonella* can invade the spleen and liver by disrupting gut integrity, leading to inflammation in these organs [50].

The present study found that *Artemisia argyit* essential oil significantly improved growth performance, as evidenced by increased BW and ADG. Key indicators of intestinal health, mucosal barrier function, permeability, and healing include intestinal morphology (such as villus height, crypt depth, and V/C ratio), intestinal epithelial tight junction proteins (including claudin, occludin, ZO-1, etc.), bacterial colonisation, and the secretion of intestinal inflammatory factors [51]. Our investigation of these parameters revealed that *S. pullorum* infection led to enhanced colonisation, intestinal damage, and an increased inflammatory response, consistent with previous studies [52].

Furthermore, high-throughput 16S rRNA sequencing demonstrated a significant improvement in the balance and diversity of gut microbiota in the intestines of chicks fed *Artemisia argyi* essential oil. It has been shown that a reduction in the number of intestinal Firmicutes (such as *Bacillus* and *Lactobacillus*) is closely associated with intestinal damage and poor growth in *S. pullorum*-infected chicks [53]. Interestingly, FMT of gut microbes from *Artemisia argyit* essential oil donors helped rehabilitate intestinal damage in the infected chicks.

Collectively, these findings suggest that *Artemisia argyit* essential oil can alleviate *S. pullorum* infection and intestinal inflammation in chicks by modifying the composition of the gut microbiota.

The intestinal tract of chicks is home to thousands of organisms, forming a complex microbiome. Dysbiosis in gut microbiota can have detrimental effects on the metabolism, immunity, and overall health of the host [54]. To investigate the impact of *Artemisia argyit* essential oil on gut microbiota, 16S rRNA gene sequencing was performed. Compared to the NC group, significant changes in the levels of *Lactobacillus* and *Parabacteroides* were found in the EOH group.

*Lactobacillus* is noteworthy for its role in defending against pathogenic microorganisms, regulating inflammation, managing gut microbiota, and preventing bacterial infections, along with its recognised safety profile [55]. Studies have indicating that feeding *Lactobacillus* can improve gut microbiota in chicks [56]. Specifically, *Lactobacillus* species such as *L. reuteri*, *L. casei,* and *L. plantarum* have all shown protective effects in chicks infected with *S. pullorum-*infected chicks [57–59], which is consistence with our findings. Probiotics from the *Lactobacillus* genus, particularly *L. reuteri*, are well-regarded for their excellent probiotic properties. Therefore, this study suggests that *Artemisia argyi* essential oil may alleviate *S. pullorum* infection in chicks by enhancing the presence of *L. reuteri*.

To understand how *Artemisia argyi* essential oil affects *S. pullorum* infections by altering gut microbiota with *L. reuteri*, we analysed the gene expression related to the proliferation and differentiation of chick intestinal epithelial cells. The results showed that *Artemisia argyi* essential oil significantly increased PCNA mRNA levels in the chick ileum, which serves as a concrete marker of cell proliferation and correlates positively with the number of crypt cells [41]. When pathogenic bacteria threaten the intestine, the intestinal mucosa functions as a barrier [60]. Our findings align with observations of changes in ileum villus height and crypt depth in chicks, indicating that supplementation with *Artemisia argyi* essential oil enhances the proliferation and maturation of the ileum epithelium during *S. pullorum* infection.

Moreover, the Wnt/β-catenin signalling is crucial for intestinal development and maintaining intestinal microenvironmental homeostasis, with its dysregulation linked to intestinal injury [61]. The activation of Wnt/β-catenin signalling is associated with the downregulation of Notch genes (Notch1, Dll1, and Hes1), which promotes the differentiation of chick intestinal epithelial cells, in line with previous research [62]. Additionally, it has been reported that *Artemisia argyi* essential oil enhances the expression of endogenous antimicrobial peptides, which contribute to various biological activities such as immunomodulation and protecting the intestinal barrier [63].

While our study indicates that *Artemisia argyit* essential oil may alleviate *S. pullorum* infection progression by increasing *Lactobacillus* abundance, we recognise several limitations. Besides *Lactobacillus,* which was the focus of this investigation, there are other beneficial intestinal microorganisms likely have unique mechanisms of resistance to *S. pullorum* yet to be explored.

**Figure 8:**
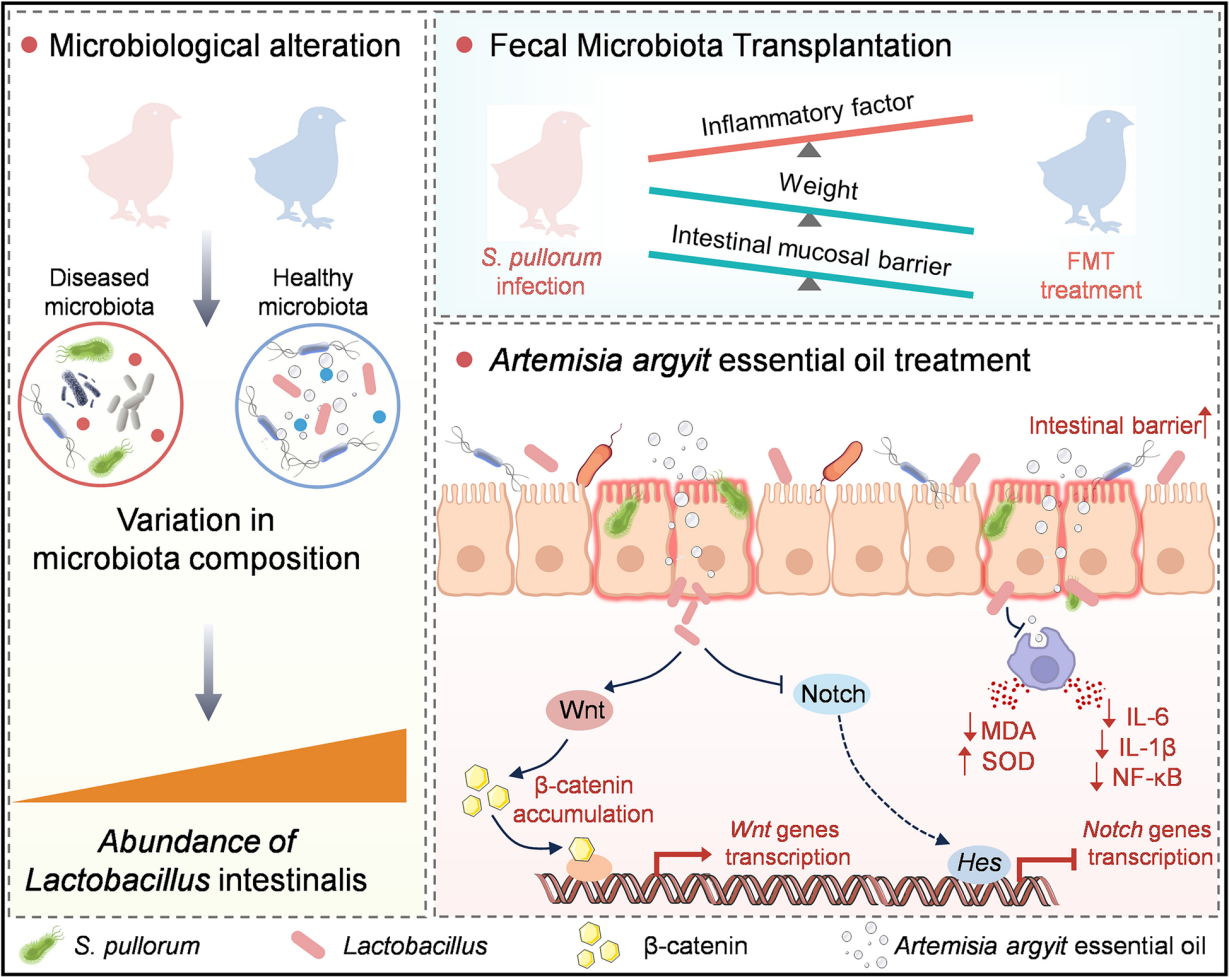
**Potential mechanisms underlying the prevention effect of** ***Artemisia argyi*** **essential oil against* S. pullorum*** **infections.**

Our results indicate that supplementation with *Artemisia argyi* essential oil alleviated intestinal mucosal damage and substantial intestinal inflammation caused by *S. pullorum* infection by regulating the gut microbiota (Figure 8). Notably, the relative abundance of *L. reuteri* in the gut microbiota increased after supplementation. Moreover, with *L. reuteri* supplementation demonstrated the ability to activate the Wnt/β-catenin pathway and improve intestinal barrier function in chicks infected with *S. pullorum*. This suggests that targeting this pathway could be crucial in preventing intestinal infections caused by *S. pullorum*. Overall, these findings provide a mechanistic explanation for the effectiveness of *Artemisia argyi* essential oil against *S. pullorum* infection, highlighting potential therapeutic strategies for managing pullorum disease.

## Supplementary Information

**Additional file 1. The primers for qPCR assays.**

**Additional file 2. Effects of supplementation *Artemisia argyi*** **essential oil on the Alpha-diversity indices of caecal microbiota communities of the** ***S. pullorum*-infected chicks. A** Chao1 index, **B** Simpson index, **C** Shannon index, **D** Observed_otus index, and **E** Goods_coverage index (*n* = 6). NC: Uninfected chicks, SP: *S. pullorum*-infected chicks, EOH: 100 mg/kg/day of *Artemisia argyi* essential oil and *S. pullorum*-infected chicks. Data are expressed as the mean ± SD. ns, *P* > 0.05.

**Additional file 3.PICRUSt2 metagenome and Heatmap inference analysis based on 16S rRNA dataset of intestinal microorganisms after** ***Artemisia argyi*** **essential oil treatment. A** Heatmap analysis of microbial taxonomic composition at the genus level. **B** Prediction of significant KEGG pathways that were different in the *Artemisia argyi* essential oil supplementation group compared to the NC group.

## Data Availability

The data analysed during the current study are available from the corresponding author on reasonable request. Part of the material in graphic was modified from Servier Medical Art, licensed under a Creative Common Attribution 4.0 Unported License.

## Abbreviations

ADG: average daily gain
ASV: amplicon sequence variants
ADFI: average daily feed intake
ASS1: arginosuccinate synthetase 1
Ada: adenosine deaminase
Axin2: axis inhibition protein 2
CMC-Na: Carboxymethyl cellulose
CFU: Colony forming units
Dll1: delta-like 1
DSS: dextran sulphate sodium
ELISA: enzyme linked immunosorbent assay
FABP-6: fatty acid-binding protein 6
F/G: feed/gain ratio
FMT: faecal microbiota transplantation
GH: growth hormone
Gleb: β-galactosidase
GLP-2: glucagon-like peptide 2
H&E: haematoxylin and eosin
HSP70: heat shock protein 70
Hes1: hairy and enhancer of split 1
IGF-1: insulin-like growth factor-1
IL-6: interleukin-6
IL-10: interleukin-10
IL-1β: interleukin-1β
KEGG: Kyoto encyclopaedia of genes and genomes
LDH: lactate dehydrogenase
*L. reuteri*: *Lactobacillus reuteri*
Lrp5: lipoproteins receptor related protein 5
LB: Luria–Bertani
LDA: linear discriminant analysis
LEfSe: linear discriminant analysis effect size
MDA: malondialdehyde
MUC-2: mucin-2
MRS: De Man, Rogosa and Sharpe
MDR: multi drug resistance
NF-κB: nuclear factor kappa-B
OTU: operational taxonomic unit
OLFM4: olfactomedin 4
PCoA: principal coordinate analysis
PCNA: proliferating cell nuclear antigen
PICRUSt: phylogenetic Investigation of Communities by Reconstruction of Unobserved State
PBS: phosphate buffered saline
*S. pullorum*: *Salmonella pullorum*
SOD: superoxide dismutase
SGLT-1: sodium-dependent glucose transporter 1
TCM: Traditional Chinese M edicine
V/C: villus height to crypt depth ratio
XLD: xylose lysine deoxycholate
ZO-1: zonula occludens-1
16S RNA: 16S ribosomal RNA
