# Supplementary material for: Ingestion of Artemisia argyit essential oil combats Salmonella pullorum infections by altering gut microbiota composition in chicks
Source: Vet Res. 2025 May 6;56:98. doi: 10.1186/s13567-025-01527-7 (PMC12057167; doi:10.1186/s13567-025-01527-7)
Supplement: Supplementary file 1 — Additional file 1. The primers for qPCR assays. [file 13567_2025_1527_MOESM1_ESM.docx]

**Additional file 1. The primers for qPCR assays**

| **Gene** | **Primer sequence (5′ to 3′)** |
| --- | --- |
| *ZO-1* | F: CCAAAGACAGCAGGAGGAGA  R: TGGCTAGTTTCTCTCGTGCA |
| *Occludin* | F: TCATCCTGCTCTGCCTCATCT  R: CATCCGCCACGTTCTTCAC |
| *MUC-2* | F: CATTCAACGAGGAGAGCTGC  R: TTCCTTGCAGCAGGAACAAC |
| *Claudin-1* | F: GAGGATGACCAGGTCAAGAAG  R: TGCCCAGCCAATGAAGAG |
| *Notch1* | F: CACCGAGGATGTGGATGAG  R: CATTGACGCAGACGCAGT |
| *Hes1* | F: GAAGTCCTCCAAACCCATCA  R: CCAGAGACACCAACCCAACT |
| *Dll1* | F: TCGTTGATTTCAATCTCGCAGC  R: GCCGCACCTGGATTTACCTC |
| *Wnt3a* | F: TTCCAGCTTCATTGTTGTGC  R: AGTGAGGACGTGGAGTTTGG |
| *β-catenin* | F: GAAGGTGGGTGCAACAGTTT  R: CTCACCAGCAGACATCAGGA |
| *Axin2* | F: GGGCTGGGGAGCTTAAAAGT  R: TCACTATCGTTTGCGCTGGT |
| *Lrp5* | F: CCGCTCTGCATTCACTACAA  R: ACCTGTGAGGCCACAAATTC |
| *HSP70* | F: TGTTCAGCTCTTTGCCATTG  R: TTGATAAGGGCCAGATCCAG |
| *GLP-2* | F: TGTAGCGCTTGGTGTTGATG  R: ACCTTCACCAGCGACTTCAC |
| *SGLT-1* | F: TTCCTCTTCCTCCTTGCTCA  R: GTGAAGACCCAGGATGCCTA |
| *FABP-6* | F: ATTAGTCGTGGTGCGTCCTC  R: CAAGATCGAAATGGGAAGGA |
| *OLFM4* | F: CAAGAGCGTTGTGGCTATCA  R: CTCTGGATGACCACGGAAGT |
| *PCNA* | F: AGCCTTCTTGCTGGTCTTCA  R: TGCAGATGTTCCTCTCGTTG |
| *Ass1* | F: ACACCTTCTTAGCCCCCAGT  R: CTGAAGGAGCAGGGCTACAC |
| *Gleb* | F: ACTTGCCCCTTTCAAGGACT  R: CAAAGCTTCCGAAAAACTGC |
| *Ada* | F: GGCCACCACAGAGTTGTTTT  R: CGCATCAAAGCCAGGTCTAT |
| *β-actin* | F: GAGAAATTGTGCGTGACATCA  R: CCTGAACCTCTCATTGCCA |
