# Supplementary figures and images for: Ingestion of Artemisia argyit essential oil combats Salmonella pullorum infections by altering gut microbiota composition in chicks
Source: Vet Res. 2025 May 6;56:98. doi: 10.1186/s13567-025-01527-7 (PMC12057167; doi:10.1186/s13567-025-01527-7)

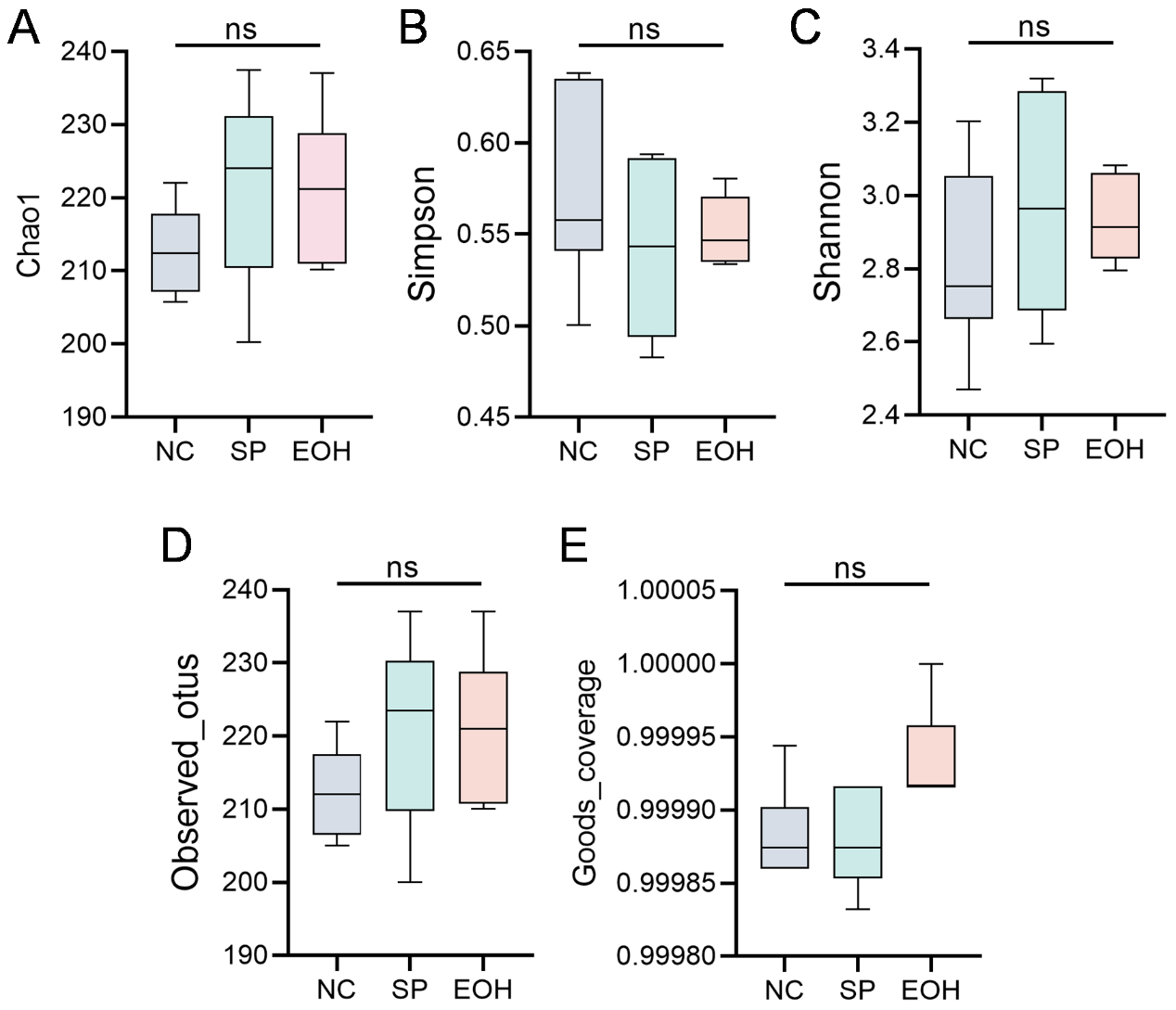

Supplement: Supplementary file 2 — Additional file 2. Effects of supplementation Artemisia argyi essential oil on the Alpha-diversity indices of caecal microbiota communities of the S. pullorum-infected chicks. A Chao1 index, B Simpson index, C Shannon index, D Observed_otus index, and E Goods_coverage index (n = 6). NC: Uninfected chicks, SP: S. pullorum-infected chicks, EOH: 100 mg/kg/day of Artemisia argyi essential oil and S. pullorum-infected chicks. Data are expressed as the mean ± SD. ns, P > 0.05. [file 13567_2025_1527_MOESM2_ESM.docx]

**
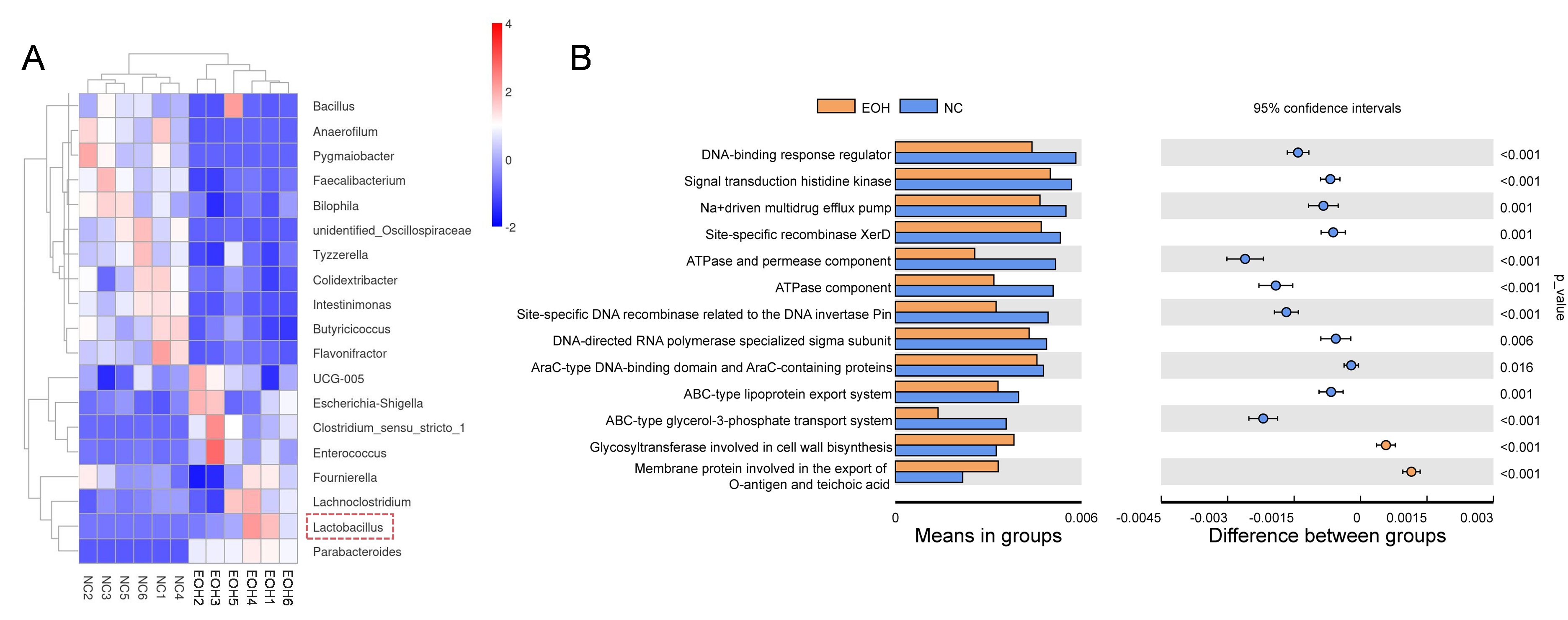
**

Supplement: Supplementary file 3 — Additional file 3.PICRUSt2 metagenome and Heatmap inference analysis based on 16S rRNA dataset of intestinal microorganisms after Artemisia argyi essential oil treatment. A Heatmap analysis of microbial taxonomic composition at the genus level. B Prediction of significant KEGG pathways that were different in the Artemisia argyi essential oil supplementation group compared to the NC group. [file 13567_2025_1527_MOESM3_ESM.docx]
